# Developing and pretesting a new patient reported outcome measure for paediatric Chronic Fatigue Syndrome/ Myalgic Encephalopathy (CFS/ME): cognitive interviews with children

**DOI:** 10.1186/s41687-019-0156-8

**Published:** 2019-11-09

**Authors:** Roxanne M. Parslow, Alison Shaw, Kirstie L. Haywood, Esther Crawley

**Affiliations:** 1 Centre for Academic Child Health (CACH), Bristol Medical School, University of Bristol, 1-5 Whiteladies Road, Bristol, BS8 1NU UK; 2 Centre for Primary Care Research, Bristol Medical School, University of Bristol, Canynge Hall, Bristol, BS8 2PS UK; 3 Warwick Research in Nursing, Division of Health Sciences, Warwick Medical School, University of Warwick, Coventry, CV4 7AL UK

**Keywords:** Chronic fatigue syndrome/ Myalgic encephalopathy (CFS/ME), Children, Patient reported outcome measure (PROM), Qualitative, Cognitive interviews, Three step test interview (TSTI)

## Abstract

**Background:**

There is a lack of patient derived, child specific outcome measures to capture what health outcomes are important to children with Chronic Fatigue Syndrome/ Myalgic Encephalopathy (CFS/ME). We developed a new Patient Reported Outcome Measure (PROM) for paediatric CFS/ME through qualitative research with children. This study aimed to pre-test the new measure through cognitive interviews with children with CFS/ME.

**Methods:**

Cognitive interviews were undertaken in children’s homes or over Skype. The Three-Step Test-Interview (TSTI) method was used to assess the quality of the draft PROM with children with CFS/ME to identify problems with initial content and design and test modifications over subsequent interview rounds. Children were purposively sampled from a single specialist paediatric CFS/ME service in England.

**Results:**

Twenty-four children and their parents took part. They felt the new measure captured issues relevant to their condition and preferred it to the generic measures they completed in clinical assessment. Changes were made to item content and phrasing, timeframe and response options and tested through three rounds of interviews.

**Conclusions:**

Cognitive interviews identified problems with the draft PROM, enabling us to make changes and then confirm acceptability in children aged 11–18. Further cognitive interviews are required with children 8–10 years old to examine the acceptability and content validity and provide evidence for age related cut offs of the new PROM to meet FDA standards. This study demonstrates the content validity of the new measure as relevant and acceptable for children with CFS/ME. The next stage is to undertake a psychometric evaluation to support the reduction of items, confirm the structure of the PROM and provide evidence of the data quality, reliability and validity.

## Background

Health Related Quality of Life (HRQoL) in children with Chronic Fatigue Syndrome/Myalgic Encephalopathy (CFS/ME) is lower than children with asthma, type 1 diabetes mellitus or junior idiopathic arthritis [1–3]. Children with CFS/ME experience a range of physical and cognitive symptoms alongside extreme fatigue [4–6] that impact on their physical function [7], social functioning at school and with friends [3, 8, 9] as well as psychological wellbeing, increasing depression and anxiety [10–12]. Measuring HRQoL through Patient Reported Outcome Measures (PROMs) is important to describe the impact of an illness on a patient’s daily functioning. PROMs provide evidence about the benefits of treatment in clinical and research settings and improve clinical decision making [13–17].

The first key stage in PROM development [17–19] is the content validity phase, during which qualitative methods are used to provide evidence that domains measured in the final instrument are important to patients [17, 19, 20]. Traditionally, child-specific PROMs have failed to incorporate children’s perspectives, instead relying on input from health professionals or parents [21–23]. The resultant measures may therefore miss outcomes that are important to children, risking content validity and measure responsiveness [24, 25]. The International Society for Pharmacoeconomics and Outcomes Research (ISPOR) taskforce advocates children as “effective content experts” [26]. There is currently no validated paediatric CFS/ME-specific PROM [27] informed by children’s views.

We sought to address this gap by developing a PROM through extensive qualitative work with 46 children with CFS/ME [28, 29] and specialist paediatric CFS/ME clinicians [30]. A child-specific conceptual framework of HRQoL was developed and provided a basis for the operationalisation of questionnaire items based on the most important outcomes to children with CFS/ME (*reference currently under review*).

A second key stage in PROM development is pretesting a new measure to ensure the final PROM is acceptable and easily understood by patients [19, 20, 31]. Guidance recommends the use of cognitive interviews to ensure “vocabulary level, item content, recall period, response options, instructions and comprehensiveness are appropriate for the target age group” [26]. Children may not interpret items the way adults intend [32] and there is a need to examine if a child can read, comprehend and respond to questionnaire items [26, 33]. This is particularly important for children with CFS/ME who often experience cognitive impairment [34–37]. Cognitive interviewing focuses on the cognitive processes that respondents use to answer questions [38], with the aim of improving the design of instruments to avoid respondent misunderstanding, minimize errors and reduce missing data [19]. The aim of the current study was to pre-test the new CFS/ME-specific PROM for children using cognitive interview methods.

## Methods

### Development of the new Paediatric CFS/ME PROM

The questionnaire was developed through a systematic approach. A conceptual framework of ‘living with paediatric CFS/ME’ developed from qualitative research (*reference currently under review*) formed the basis of the new measure with quotes taken directly from children used to construct age appropriate items for each domain of the framework [19]. Draft items (*n* = 95) were then reviewed by 22 paediatric CFS/ME health professionals from the U. K and the Netherlands in a day-long meeting. Health professionals were asked to review the items for content and wording, consider the comprehensiveness of domains and any missing outcomes, and ensure that items were clinically relevant and measured the range from mild to moderate severity. Healthy children as well as children with CFS/ME were consulted on their views on the structure and formatting of existing generic child PROMs (e.g. question tense, recall period, response options) with the new measure designed around their preferences.

A final 67 items were selected to form the basis of a draft questionnaire ready for pre-testing in cognitive interviews. The preliminary paediatric CFS/ME PROM consisted of 67 items grouped into 4 domains (symptoms, physical, social & psychological) and 11 subdomains: sleep (1 item), tiredness/fatigue (9 items), cognitive difficulties (4 items), individual symptoms (7 items), fluctuation and payback (3 items), physical function (daily activities and mobility) (11 items), participation in school life (3 items) participation in social life (7 items), mood (10 items), anxiety (7 items) and self-esteem (5 items). Items on physical function and participation were developed to capture the range of severity described by children from mild (problems with outdoor, sustained activities and sports but attending full time school) to moderate (significant physical disability, problems washing, can only do indoor activities and attending part time school) to optimise the relevance and coverage of the PROM [19, 39]. Negatively phrased items (“I had problems remembering things”) were used to assess ill-being (problems/symptoms) [40] whereas items on social participant and self-esteem were positively phrased (“I felt good about myself”) to balance the questionnaire [41]. The questionnaire was developed as a self-report instrument for children 8–18 years old to report how they have been feeling ‘over the past week’ using five point Likert response scales that children have found easier to complete [26, 42] targeting: severity (Not at all- Very much), frequency (Never-Always) and interference (With no difficulty- Not able to do).

### Pretesting the new Paediatric CFS/ME PROM (cognitive interviews)

#### Study design

Three rounds of cognitive interviews were undertaken with changes made to the questionnaire after each round and tested in an iterative process with different children in subsequent rounds until saturation (Round 1 *n* = 10. Round 2 *n* = 9; Round 3 *n* = 5). Changes made to the questionnaire in round 1 were tested in round 2 and then a further 5 children were recruited for a final round to check the final changes made in round 1 and 2 were acceptable. No significant findings emerged in round 3. The Three-Step Test-Interview (TSTI) method was used to assess the quality of the new self-complete questionnaire [43]. The TSTI combines think aloud and verbal probing/debriefing in a sequence of stages: 1) Think aloud (verbalizing thoughts whilst completing a measure) by participants and observation of response behaviour by the interviewer to collect primary data on any problems with the measure (e.g. skipping questions; hesitation, changing response options); 2) follow-up probing by the interviewer to explore any observations e.g. “Did I hear you say.. .? ”; and 3) debriefing by the interviewer aimed at eliciting overall experiences and opinions of participants.

#### Participants

Children aged 8–18 years, diagnosed with mild to moderate CFS/ME (not housebound) [5], were recruited from a specialist paediatric chronic fatigue service in South West England. Sampling of participants was purposeful [44] and guided by participant characteristics (age, gender, and disease severity). Further children were recruited in subsequent rounds to check changes made to the questionnaire based on earlier findings were appropriate and understandable.

#### Data collection

Cognitive interviews were undertaken face to face in participants’ own homes or over Skype. While we aimed to interview children alone, parents were given the option to remain. If parents helped during the interviews, how they helped was noted. Interviews were audio-recorded using an encrypted digital recorder and written notes were made.

*Step1:* Participants completed the PROM as they would do normally, whilst encouraged to verbalize what they were thinking and why they selected their responses. Children were given an example of think aloud at the start to help demonstrate what was required. The interviewer intervened as little as possible and recorded the processes that participants used in arriving at an answer as well as any difficulties (reading difficulty, pausing, flipping pages).

*Step 2:* The interviewer followed up on observations they noted down that were unclear using spontaneous probes (e.g. “Did I hear you say?”).

*Step 3:* The interviewer followed a topic guide with non-leading probes to ensure comparability across interviews. Probes were based on Tourangeau’s four-stage model [45] to explain how information is understood, retrieved and organized by respondents trying to answer questions and covered each element of the questionnaire: layout, instructions and any issues children felt were missing from the questionnaire (Table 1).

**Table 1:** Extract of cognitive probes from topic guide

Cognitive/ Questionnaire Component	Interview Probe
Follow up on observations	Why did you pause on this question?
Comprehension/ Items	What does [item content] mean to you?
Retrieval/ Timeframe	What did you remember when you read this question?
Judgement	Describe your experiences with [concept] over the (timeframe).
Response	How did you select your [response option?]
Overall feedback	Are there things that we forgot to ask about that you think are important? What do you think about the questionnaire?

#### Data analysis

Audio recordings were reviewed in detail by RP, alongside the written notes, and data inserted on a structured excel form that had rows for each questionnaire item and individual participants and columns for participant responses to each element of the questionnaire (e.g. item wording, timeframe, response options etc. …). This ensured that for each questionnaire item, we recorded information from observation of questionnaire completion, participants’ feedback on item meaning, and any difficulties and suggestions for change. For each participant, we also noted feedback on the appropriateness of the timeframe and response options, if anything was missing and general impressions of the questionnaire. A summary of each item was generated including recommended changes. Quotes were transcribed verbatim to illustrate findings or reasons for change.

##### Expert appraisal

**Fig. 1:**
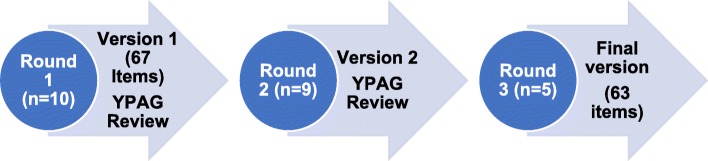
Rounds of cognitive interviewing

An iterative process was adopted with changes made to the questionnaire from the first round of interviews tested in subsequent rounds until saturation [38, 46, 47]. Findings from each interview round were reviewed with ‘experts’ (e.g. core research team and a CFS Young Persons Advisory Group (YPAG) [47, 48]. The CFS YPAG met in person after round 1 (5 attendees- 3 young people with CFS/ME, ages 15–17 years of age, one young adult and 1 parent) and were consulted by email after round 2 (3 PAG members, 15–17 years of age). The final version of the questionnaire was then taken to a group of 15 healthy children (aged 10–17, 11 girls and 4 boys) to check the general readability and comprehension of items by school aged children. Children were given the questions to complete as they would do normally and were asked to indicate if the questionnaire was easy to complete or if there were any difficulties. Children wrote their feedback on the actual questionnaires and discussed this with a facilitator (RP) (Fig. 1).

##### Questionnaire modification

The FDA guidance was followed when modifying items based on the cognitive interviews [17] (Table 2). Where misinterpretation was observed or relevance was low, this indicated that items were less acceptable and change should be considered. Problems that re-occurred over successive interviews were considered for editing and re-testing. Problems identified with younger children were considered even if they only occurred in a few interviews. Decisions to modify items were documented in an item-tracking matrix that noted the problem findings and decisions for modification in order to provide an audit trail [19] (Additional file 1). Decisions were made about whether to retain, modify, remove or add items on an item by item basis. No items were deleted from the list generated by previous qualitative studies without agreement from the CFS YPAG.

**Table 2:** Reasons for changing items during pre-testing (Adapted from [17])

Item Property	Reasons for change or deletion
Clarity of relevance	• Not relevant to a large number of the participants • Large amounts of requests for clarification from participants • Participants interpret items in a different way than intended by the conceptual framework
Response range	• High number of participants response at extreme ends of the scale • Participants feedback that the none of the response options apply to them
Variability	• All participants give the same answer • Participants do not give different responses even when important differences are known
Redundancy	• Item duplicates information collected from other items

## Results

### Participants

Twenty four children participated over three rounds of cognitive interviewing (Table 3). Children ranged in age from 8 to 18 years (mean age 13 years): 8 (8–11 year olds), 7 (12–15 year olds) and 9 (16–18 year olds). Thirteen were female (54%), 14 were mildly affected (58%) and all were white Caucasians. Most children were interviewed at home 19 (79%) and 5 (21%) using Skype. All those interviewed on Skype were aged 16–18 years old. Parents were present in 11 (46%) interviews. Parents mainly observed but tended to take part more with younger children. Nineteen children (79%) covered all domains, 5 (21%) did not complete the entire PROM: 1 ran out of time and four got tired or lost concentration. Interviews usually lasted between 40 min to an hour (mean 43 min, range 27 min-1 h 25 min).

**Table 3:** Participants taking part in cognitive interviews

Child I.D	Gender	Age at interview	Interviewed alone/parent present	Interviewed at home or Skype	Sub domains covered
CI1	Female	14	Parent present	Home	All
CI2	Female	17	Alone	Home	All
CI3	Female	15	Alone	Home	4/6
CI4	Female	16	Alone	Home	All
CI5	Female	16	Alone	Skype	All
CI6	Female	12	Parent present	Home	All
CI7	Male	11	Parent present	Home	3/6
CI8	Male	16	Alone	Skype	All
CI9	Male	17	Alone	Home	All
CI10	Female	8	Parent present	Home	3/6
CI11	Male	14	Parent present	Home	All
CI12	Male	10	Parent present	Home	All
CI13	Female	17	Alone	Skype	3/6
CI14	Female	17	Alone	Skype	All
CI15	Male	16	Parent present	Home	All
CI16	Female	14	Alone	Home	All
CI17	Male	12	Alone	Home	All
CI18	Male	18	Alone	Skype	All
CI19	Male	10	Parent present	Home	All
CI20	Female	11	Alone	Home	All
CI21	Male	11	Parent present	Home	All
CI22	Female	14	Parent present	Home	All
CI23	Female	11	Alone	Home	All
CI24	Male	10	Parent present	Home	2/6

Overall, all children preferred the new PROM to current generic measures and felt that it captured issues that are relevant and not just ‘fatigue’, “It’s a lot more relevant than the other ones [generic] were” (CI22, female, aged 14). Children liked the format with instructions in orange and alternating shades as well as the different sections covering the various aspects of health; “One of the easiest ones I’ve ever seen. Broken down which is nice.” (CI2, female, aged 17). Younger children reported more problems with specific words or longer items. Problems were identified in the following areas: content and item phrasing, timeframe and response options. Changes were made and tested in subsequent interviews (Additional file 1).

### Content and item phrasing

Based on the cognitive interviews, 12 items were removed or merged with others as they were repetitive, 26 items were found to be unclear or difficult to answer and were revised and 9 items were added to symptoms and schooling, to measure individual aspects. Children reported that there were too many items on ‘tiredness’ and ‘physical function’. They found items such as “I had trouble finishing things” too ambiguous depending on the activity and motivation. For the item “I could move around the house” most children thought of ‘going up and down the stairs’ which was included as another item. One question about ‘school attendance’ was not felt to be adequate; many children attended full time school but missed lessons or had to take breaks. As a result, three more items covering (missing school, missing lessons & problems paying attention) were added.

"You might be physically there but mentally you may not actually be there … you are not always able to fully concentrate … whether you contribute to the lesson" (CI8, male, aged 16)

Items about payback (i.e. tired after activity) resulted in a ceiling effect as use of the term ‘high energy activities’ resulted in all children selecting that they would *always* get payback. In the final round, ‘active day’ was used and this worked well to capture the payback that can be caused when children have a busy day or usually a day out:

“I went swimming on the weekend at my Dads house for 45 minutes and after I felt really bad “(CI23, female, aged 11)

Minor revisions were made to the ‘participation’ and ‘emotional wellbeing’ domains. Examples were modified to make them more relevant (e.g. ‘board games’ replaced with ‘playing and going on the computer’). ‘Sad’ was amalgamated with ‘feeling down’. The item “I felt like I’m being left behind” was felt to be ambiguous thus was amended to: “I worried about being able to do what other people my age can do”. In subsequent rounds, it demonstrated its applicability across the age range as younger children referred to ‘not being able to do physical activities with friends’ and older children thought about life stages such as university.

#### Age differences

Some children felt the examples given of social activities were not applicable to them, therefore, the content of the school and social participation domains were adjusted and different versions developed for 8–15 & 16–18 year olds and tested in later interviews. For example, ‘leisure activities’ was used for 16–18 years olds rather than ‘afterschool clubs’ for 8–15 year olds. Items were also edited to refer to “school, college or work/apprenticeship” for 16–18 year olds.

### Timeframe

In the first round, children found ‘the past week’ recall period difficult as some may have had a good or bad week and felt it was not representative of how they were at the moment. A two-week timeframe was introduced in round two and more children preferred it:

“Maybe two weeks, people can remember that and it gives you the longer timespan to remember things and give a lot more solid answers”. (CI2, female, aged 17)

“Int: So if I asked about 2 weeks do you think that would be better. Do you think you would remember?

CI6: Yeah

Int: What if it asked about how you are at the moment?

CI6: you would think how you were that day to be honest. So you might have a good day and you might have a bad day." (CI6, female, aged 12)

### Response options

In round one and two, children had difficulty answering items in the participation domain as most are ‘able to do activities’ (e.g. go to the park or meet friends) but would get payback (e.g. tired after activity) which was not captured in the frequency response options (never-always).

“I can do it. I would struggle, I usually push through things” (CI6, female, aged 12).

“I don’t see it as ‘being able to do it’ as such, if I was able to do it I’d do it and not crash and burn, that’s the difficult thing about answering these, I could probably play a board game but the effect would be a few days afterwards.” (CI2, female, aged 17)

"I love football and cricket and swimming and I won’t be able to do that for as long as I want" (CI23, female, aged 11)

Parents of younger children also commented that their child could do almost anything but would require breaks and pacing. Comments- this appears bold on the printed PDF. Please unbold.

"CIP7: He can do anything he wants to do but it’s the amount he has to restrict. Anything is possible, but it depends on the chunks, he can do it but he can only do a few minutes a time" (CIP7, mother of male aged 11)

The ‘frequency’ response options were changed to ‘interference’ (with no difficulty- with a lot of difficulty) options for the participant domain and these worked well in the last round of cognitive interviewing:

"I think these are quite good [difficulty response options] because they have the word 'difficulty' in, so it’s like 'how difficult do you find this to do', not just 'can you do this or not'" (CI14, female, aged 17)

"I: When you think about 'difficulty' what are you thinking about?

CI6: Just struggling or thinking 'I'm shattered' while doing it". (CI6, female, aged 12)

#### School response options: days changed to hours

Some children struggled to indicate their school attendance in days as many moderately affected children only attended school for a few hours a day:

"By full time you mean 5 days but for me full time is 6 hours each day". (CI5, female, aged 16)

The response options were edited to hours with prompts. This worked well across the older and younger age groups in round 2 and 3:

"I like how you've put 'one day a week', because it doesn’t make sense if you just put 1-7 hours" (CI22, female, aged 14)

Cognitive testing and review by the YPAG resulted in the final measure ready for psychometric testing: 63 items grouped into 4 domains, 11 subdomains: sleep (1 item), tiredness/fatigue (6 items), cognitive difficulties (4 items), individual symptoms (8 items), fluctuation and payback (3 items), physical function (daily activities and mobility) (8 items), participation in school life (6 items) participation in social life (7 items), mood (7 items), anxiety (8 items) and self-esteem (5 items). It was developed as a self-report instrument with patients reporting how they have been feeling ‘over the past two weeks’ on 5 point response scales: severity (Not at all- Very much), frequency (Never-Always) and interference (With no difficulty- Not able to do).

## Discussion

The cognitive interviews were valuable to identify problems with the initial content and design of the draft PROM including: 1) repetitive items for tiredness and missing items on school participation, 2) items on payback requiring rewording and age appropriate examples required for participation items, 3) unsuitable timeframe with most children preferring 2 weeks and 4) the value of the interference response options. Rounds of interviews enabled us to make these changes and confirm the acceptability of the questionnaire in children aged 11–18.

### Strengths and weaknesses

Children’s (*n* = 46) verbatim quotes were used to craft items, grounding the new PROM on children’s expressed concerns and preserving children’s speech for strong internal and external validity [26]. To ensure optimal content validity, the perspectives of children and health professionals were used to generate, format and refine the draft PROM, ensuring items were included that mattered to patients but were also important clinically [49]. A range of paediatric CFS/ME health professionals were recruited from around the U.K as well as from the Netherlands to review the draft PROM. This increases the applicability of the new PROM and ensures that perspectives from different specialist paediatric CFS/ME services were included.

The cognitive interviews have been an extremely valuable step in developing our new PROM and optimising the sensitivity of the instrument. For example, as a result of feedback from children, the response options in the participation domain were changed to interference (with no difficulty- not able to do) to capture that children with CFS/ME can often participate in ‘normal’ activities but they will then experience payback or increase in their symptoms and difficulty as a result of taking part. We believe this is one of the first studies to use the Three Step Test Interview (TSTI) in children. There are studies available in the literature that have used the TSTI for adult PROMs [50–53]. The TSTI includes the traditional methods of think aloud and verbal probing in sequential steps and this worked well as if a child was capable of ‘think aloud’ less verbal probing was required and vice versa. The cognitive debriefing/probing method has traditionally been used in studies developing new PROMs in children: DISABKIDS [54], Haemo-QoL [55], EQ. 5D-Y [56] and PedsQL disease specific modules [57] as well as the ‘think aloud’ technique [33, 58–61]. Some younger children found both think aloud and debriefing methods difficult. In these cases, asking them to look at the questions and physically mark with a pen/pencil the words or items they found difficult worked more effectively. Two age appropriate topic guides may be needed to draw on the different cognitive interview methods for different ages. Further empirical evidence is required to explore the TSTI in children.

The majority of children (*n* = 19) reviewed the whole questionnaire, however, four got tired or lost concentration. The interviews took an average 43 min and we feel this is a much longer process, than asking a child to complete a questionnaire normally without incorporating think aloud and probing. Therefore, future research should divide long questionnaires up between participants and recruit a larger sample size to ensure the interviews are more manageable for children, particularly those with cognitive impairment. We ended interviews after 24 participants had been recruited as no new significant findings were emerging, fulfilling the 10–15 interviews per item recommended sample size by some researchers [38].

The ISPOR task force recommend an adequate sample size “at the upper and lower bounds of the target age range” [26]. We had problems recruiting younger children (< 11 years old) as fewer younger children attend the specialist paediatric CFS/ME clinic. CFS/ME is more common in adolescents and girls [62–70] and therefore, there is good evidence for content validity of the new PROM in children who more commonly get CFS/ME. However, ensuring that the new PROM is age appropriate across the 8–18 age range was a key challenge in this study. The cognitive abilities of an 8 year old can differ substantially from a 10 year old [71]. Thus, further cognitive interviews with younger children (< 11 years old) are required to provide robust evidence for age related cut offs of the new PROM to meet FDA standards [17, 26]. Following this, the next stage of psychometric testing should ensure children younger than 11 years old are sufficiently represented. The literacy level of participants was not recorded and this may have been useful ensure the items were understandable to a wide range of respondents [19, 72].

### Results in context with previous literature

The results from this study are consistent with other cognitive interview studies for children’s PROM development. We found children had difficulty understanding ambiguous terms such as “activities”. In cognitive interviews involving 77 children (8–17 years of age) to edit items of the PROMIS paediatric item bank [73], problematic terms included ‘social activities’. Our study revealed that children with CFS/ME found interference response options (With no difficulty- Not able to do) particularly helpful to describe how they are able to do an activity but this may be ‘difficult’ and result in payback (e.g. tired after activity) which is a key feature of CFS/ME. These response options may be applicable in other fatigue measures.

As a result of the cognitive interviews, the recall period of the new measure was changed from 1 week to 2 weeks. Children 8 years and older are thought to be able to recall a 4 week period [74]. This is consistent with well-known child-report measures such as the Child Health Illness Profile [75], Child Health Questionnaire [76] and the PedsQL [77] with a recall period of the past month and are widely used in clinical trials [26]. Shorter recall periods may fail to capture symptoms or events that occur outside the specified period or may misrepresent a particular experience [78]. This was clear in the cognitive interviews as CFS/ME fluctuates and children felt a shorter period did not represent how they were generally feeling. Interventions in CFS/ME usually occur over months [79–81] and children are expected to make small changes gradually. Whilst fluctuation is important (and captured in items within the PROM), health professionals are interested in sustained change (either worse or better) rather than the experience of the last few days and therefore it is important to capture long term changes to patient status rather than short term fluctuation. Although the FDA do not specify an optimal recall period, shorter recall periods are preferred in children [26]. Therefore, this 2-week recall may be problematic and requires further consideration in the future development of the measure.

We developed two different versions of the questionnaire for 8–15 & 16–18 year olds. School or college attendance (including missing lessons, taking breaks and problems concentrating) is one of the most important outcomes for children with CFS/ME (***reference currently under review***). Minor modifications were made to the school and social participation domains based on feedback from the cognitive interviews with differences between those in school (8–15) versus those more likely to be in college or work (16–18). Further work needs to be done with younger children (< 11 years old) to decide if a further version is needed.. This is consistent with the well-known generic child measure PedsQL, which has the same overall domains but different formatting for the various age groups 5–7, 8–12, and 13–18 years [82]. A recent systematic review of generic multi-dimensional PROMS used for children up to age 18 [83] demonstrated that the majority (29 out of 35) of child PROMs have the same version across the age range [84, 85]. However, some condition specific child measures include multiple versions: the Childhood Asthma Questionnaire (4–7, 8–11 & 12–16 years of age) [86] and the Paediatric Advanced Cancer-Quality of Life (PAC-QoL) scale has two versions (8–12 & 13–18) to account for developmental differences [87]. Other generic child measures such as the PROMIS paediatric item bank [88] and KIDSCREEN [74, 89, 90] have the same forms for ages 8–18 years, and the Oxford Foot and Ankle PROM [91] has the same version for ages 5–15. This allows for the longitudinal use of the questionnaire and comparison of results across age groups [92].

## Conclusions

This study described the careful development of a long list of items for the draft child specific CFS/ME PROM grounded in a child specific conceptual framework. Items were reviewed and refined through clinical expert review as well as healthy children and paediatric CFS/ME patient groups. The measure was then tested through cognitive interviews which illuminated problems, allowing changes and confirming acceptability in children aged 11–18. We were unable to confirm acceptability in younger children (< 11 years old) and further cognitive interviews are required with children 8–10 years old to examine the acceptability and content validity and provide evidence for age related cut offs of the new PROM to meet FDA standards. The long form questionnaire is currently too long to be readily completed in routine practice or research settings. The next essential step is a psychometric evaluation in the target population. This will support the reduction of items, confirm the structure of the PROM and provide initial essential evidence of the data quality, reliability and validity.

## Supplementary information

**Additional file 1.** Summary of changes to items based on cognitive interviewing rounds and YPAG consensus.

## Data Availability

The datasets used and/or analysed during the current study are available from the corresponding author on reasonable request.

## Abbreviations

CFS: Chronic Fatigue Syndrome
HRQoL: Health-related quality of life
ME: Myalgic Encephalopathy
PROM: Patient-reported outcome measure
YPAG: Young persons advisory group
