# Supplementary material for: Developing and pretesting a new patient reported outcome measure for paediatric Chronic Fatigue Syndrome/ Myalgic Encephalopathy (CFS/ME): cognitive interviews with children
Source: J Patient Rep Outcomes. 2019 Nov 9;3:67. doi: 10.1186/s41687-019-0156-8 (PMC6842364; doi:10.1186/s41687-019-0156-8)
Supplement: Supplementary file 1 — Additional file 1. Summary of changes to items based on cognitive interviewing rounds and YPAG consensus. [file 41687_2019_156_MOESM1_ESM.docx]

Additional file 1. Summary of changes to items based on cognitive interviewing rounds and YPAG consensus

| **Item at Pre-Test** | **Round 1** | **Round 2** | **Round 3** | **Final Item** |
| --- | --- | --- | --- | --- |
| I felt tired (e.g. worn out or drained or heavy or weak) | No change | No change | No change | Original retained |
| I had pain | Change | No change | No change | I hurt or had pain (e.g. headache or sore throat or body pain) |
| I ached | No change | No change | No change | Original retained |
| I had one or more symptoms (e.g. headaches, pain, sore throat, problems concentrating, feeling dizzy or sick) | Change | N/A | N/A | Item removed. See new items below. |
| ADDED: I felt sick or nauseous | N/A | No change | No change | Added: I felt sick or nauseous |
| ADDED: I felt dizzy | N/A | No change | No change | Added: I felt dizzy |
| ADDED: I felt unwell or poorly | N/A | Change | No change | Added: I felt unwell with my Chronic Fatigue Syndrome/ME |
| I had difficulty sleeping (e.g. getting to sleep or waking up a lot) | Change | No change | No change | I had difficulty sleeping (e.g. getting to sleep or staying asleep) |
| I found it hard to wake up and get up in the morning | No change | No change | No change | Original retained |
| I was so tired I was in bed or in a chair most of the day | Change | No change | No change | I was so tired I was in bed or on the sofa most of the day |
| I felt like I was going to fall down | Change | Change | No change | I was so tired I felt like I needed to stop what I was doing |
| I got tired easily | No change | No change | No change | Original retained |
| I had trouble starting things | Change | N/A | N/A | Item removed |
| I had trouble finishing things | Change | N/A | N/A | Item removed |
| I had little energy to do things | No change | Change | No change | I felt too tired to start things |
| I was too tired to do things I enjoy | No change | Change | N/A | Item removed |
| I found it hard to concentrate or pay attention to things | No change | No change | No change | Original retained |
| I found it hard to read or do homework | Change | N/A | N/A | Item removed |
| I had problems remembering things | No change | No change | No change | Original retained |
| I found it hard to think quickly | Change | No change | Change | I found it hard to think |
| I found it hard to eat regular meals | Change | No change | No change | I found it hard to eat |
| I had problems travelling on the school bus or in a car | Change | N/A | N/A | Item removed |
| ADDED: I found it hard to pay attention when talking to people | N/A | N/A | No change | Added: I found it hard to pay attention when talking to people |
| One or more symptoms stopped me doing things I wanted to do  (e.g. tiredness, problems concentrating, headaches, sore throat, body pain or aches, or feeling dizzy or sick) | No change | No change | No change | Original retained |
| I found it had to enjoy things because of one or more symptoms  (e.g. tiredness, problems concentrating, headaches, sore throat, body pain or aches, or feeling dizzy or sick) | No change | No change | No change | Original retained |
| The symptoms changed from day to day (good days and bad days) (e.g. feeling tired, problems concentrating headaches, sore throat, body pain or aches, feeling sick or dizzy) | No change | No change | No change | Original retained |
| I felt worse after doing high energy activities (e.g. walking, sport) | Change | Change | No change | I felt worse (e.g. really tired or dizzy or in pain) after having an active day |
| I was too tired to do anything the next day after high energy activity (e.g. walking, sport) | Change | Change | No change | I was too tired to do my normal activities after having an active day |
| I could get up from a chair | No change | No change | No change | Original retained |
| I could wash and dress myself | No change | No change | No change | Original retained |
| I could move around the house | No change | Change | N/A | Item removed |
| I could stand up for a long period (30 minutes) | Change | N/A | N/A | Item removed |
| I could do indoor activities (playing board game) for 30 minutes | Change | Change | N/A | Item removed |
| I could walk up a flights of stairs | No change | Change | No change | I could walk up and down stairs |
| I could take a short walk outside (10 minutes) | No change | No change | No change | Original retained |
| I could take a long walk outside (30 minutes) | No change | No change | No change | Original retained |
| I could do sports or exercise (30 minutes) | Change | Change | No change | I could do P.E or sports (30 minutes) |
| I could be out of the house all day | No change | Change | No change | I could be at school/ college or work all day |
| I used a wheelchair when going out | Change | No change | No change | I used crutches or a wheelchair when going out |
| Thinking about a typical week last term, how much school or college did you attend? One half day One day Two days Three days Four days Full time | Change | No change | No change | None About 1 day a week or 1 hour everyday 1-14 hours About 2 days a week or 2 hours everyday 15- 28 hours Part time (about 3 days a week or 3 ½ hours everyday) 29-42 hours About 4 days a week 43-56 hours Full time (about 5 days a week) 57-70 hours |
| Do you currently have home tuition? If yes, how many hours of home tuition did you have last week? Less than one hour One hour Two hours Three hours More than three hours | Change | No change | No change | Do you currently have home tuition or do an online course (16-18 year olds). If yes, how many hours did you manage in the last two weeks? None 1-3 hours 4-6 hours 7-9 hours 9 hours or more |
| ADDED: I missed school because of my Chronic Fatigue Syndrome/ME | N/A | Change | No change | Added: I missed school because of my Chronic Fatigue Syndrome/ME |
| ADDED: I missed lessons or had to take extra breaks when in school | N/A | Change | No change | Added: I missed lesson or had to take breaks when in school |
| ADDED: I found it hard to pay attention in school | N/A | Change | No change | Added: I found it hard to pay attention in school |
| I found it hard to keep up with my school or college work | No change | No change | No change | Original retained |
| I have been able to do indoor activities I enjoy (e.g. board games) | Change | No change | Change | I could do things at home I enjoy (e.g. playing/ going on the computer). |
| I have been able to do outdoor activities I enjoy (e.g. going to the park) | No change | No change | No change | I could do things outside I enjoy (e.g. going to the park/walks) |
| I have been able to do sports and exercise that other people my age can do | No change | Change | N/A | Item removed |
| I have been able to do hobbies that other people my age can do | Change | No change | No change | I could do hobbies or afterschool clubs/leisure activities that enjoy |
| I have been able to go out (e.g. to town, shopping, to the cinema) | No change | Change | No change | I could go out (e.g. to town or shopping) |
| ADDED: I could do things I enjoy for as long as I want to | N/A | No change | No change | Added: I could do things I enjoy for as long as I want to |
| I have been able to join in with my friends | Change | No change | No change | I have been able to spend time with my friends |
| I have been able to go out with my family (e.g. walks, days out) | No change | No change | No change | Original retained |
| I felt down | Change | No change | No change | I felt down or sad |
| I felt sad | Change | No change | No change | Amalgamated |
| I found it hard to enjoy things or have fun | No change | No change | No change | Original retained |
| I cried easily | No change | Change | No change | I got upset easily |
| I felt lonely | No change | No change | No change | Original retained |
| I felt left out | No change | No change | No change | Original retained |
| I felt frustrated | No change | No change | No change | Original retained |
| I felt worried | No change | No change | No change | Original retained |
| I worried about overdoing it and crashing (worse symptoms) | Change | No change | No change | I worried about doing things because I might get tired. |
| I worried if I am going to have a good or a bad day | Change | N/A | N/A | Item removed |
| I felt like I am being left behind | No change | Change | No change | I worried about being able to do what other people my age can do |
| I worried about what other people think of me | No change | No change | No change | Original retained |
| I worried about going into school or college | No change | No change | No change | Original retained |
| ADDED: I worried about being out of the house | N/A | No change | No change | Added: I worried about being out of the house |
| I worried about falling behind with my school/ college work | No change | No change | No change | Original retained |
| I worried about my future | No change | No change | No change | Original retained |
| It has been hard to motivate myself to do school or college work | Change | N/A | N/A | Item removed |
| I found it hard to get started on things | No change | Change | No change | I felt less interested in doing things I enjoy |
| I felt good about myself | No change | No change | No change | Original retained |
| I felt good about how I'm doing at school or college | No change | No change | No change | Original retained |
| I felt good about my ability to do exercise, sports or hobbies | Change | No change | No change | I felt confident to do things I enjoy (e.g. spending time with friends, hobbies) |
| I felt good about my friendships | No change | No change | No change | Original retained |
| I felt I will recover from CFS/ME | No change | Change | No change | I felt I will recover from Chronic Fatigue Syndrome/ ME |
